# Adverse Childhood Experiences, Support, and the Perception of Ability to Work in Adults with Disability

**DOI:** 10.1371/journal.pone.0157726

**Published:** 2016-07-05

**Authors:** Sophia Miryam Schüssler-Fiorenza Rose, Jessica G. Eslinger, Lindsey Zimmerman, Jamie Scaccia, Betty S. Lai, Catrin Lewis, Eva Alisic

**Affiliations:** 1 Spinal Cord Injury Service, Veterans Affairs Palo Alto Health Care System, Palo Alto, California, United States of America; 2 Department of Neurosurgery, Stanford University School of Medicine, Stanford, California, United States of America; 3 Center on Trauma and Children, Department of Psychiatry, College of Medicine, University of Kentucky, Lexington, Kentucky, United States of America; 4 Dissemination and Training Division, National Center for Posttraumatic Stress Disorders, Veterans Affairs Palo Alto Health Care System, Menlo Park, California, United States of America; 5 Department of Psychiatry and Behavioral Sciences, University of Washington, Seattle, Washington, United States of America; 6 Adler School of Professional Psychology, Chicago, Illinois, United States of America; 7 School of Public Health, Georgia State University, Atlanta, Georgia, United States of America; 8 National Centre for Mental Health, Cardiff University Institute of Psychological Medicine and Clinical Neurosciences, Cardiff, Wales, United Kingdom; 9 Monash University Accident Research Centre, Monash University, Melbourne, Australia; 10 Department of Psychosomatics and Psychiatry, University Children’s Hospital Zurich, Zurich, Switzerland; National Center of Neurology and Psychiatry, JAPAN

## Abstract

**Objective:**

To examine the impact of adverse childhood experiences (ACEs) and support on self-reported work inability of adults reporting disability.

**Participants:**

Adults (ages 18–64) who participated in the Behavioral Risk Factor Surveillance System in 2009 or 2010 and who reported having a disability (*n* = 13,009).

**Design and Main Outcome Measures:**

The study used a retrospective cohort design with work inability as the main outcome. ACE categories included abuse (sexual, physical, emotional) and family dysfunction (domestic violence, incarceration, mental illness, substance abuse, divorce). Support included functional (perceived emotional/social support) and structural (living with another adult) support. Logistic regression was used to adjust for potential confounders (age, sex and race) and to evaluate whether there was an independent effect of ACEs on work inability after adding other important predictors (support, education, health) to the model.

**Results:**

ACEs were highly prevalent with almost 75% of the sample reporting at least one ACE category and over 25% having a high ACE burden (4 or more categories). ACEs were strongly associated with functional support. Participants experiencing a high ACE burden had a higher adjusted odds ratio (OR) [95% confidence interval] of 1.9 [1.5–2.4] of work inability (reference: zero ACEs). Good functional support (adjusted OR 0.52 [0.42–0.63]) and structural support (adjusted OR 0.48 [0.41–0.56]) were protective against work inability. After adding education and health to the model, ACEs no longer appeared to have an independent effect. Structural support remained highly protective, but functional support only appeared to be protective in those with good physical health.

**Conclusions:**

ACEs are highly prevalent in working-age US adults with a disability, particularly young adults. ACEs are associated with decreased support, lower educational attainment and worse adult health. Health care providers are encouraged to screen for ACEs. Addressing the effects of ACEs on health and support, in addition to education and retraining, may increase ability to work in those with a disability.

## Introduction

In the United States, roughly 37 to 56 million people, including 11.6% of adults aged 18–64 years, live with a disability [1, 2]. Disability is associated with work inability among some individuals, but not others [3]. Over 70% of people with a disability have experienced childhood adversity which is higher than the general population [4]. Adverse childhood experiences (ACEs) have been linked with increased health risk behaviors [5], worse education outcomes [6], impaired worker performance [7], adult psychological distress [5, 8–10], worse physical and mental health [5, 11] and increased disability rates in adulthood [12]. Much less is known about how ACEs may affect the ability to work of people with disability. Increasing the understanding of risk and protective factors that may affect the self-perceived inability to work of individuals with disability is needed, since it can inform rehabilitation assessment and treatment.

**Fig 1:**
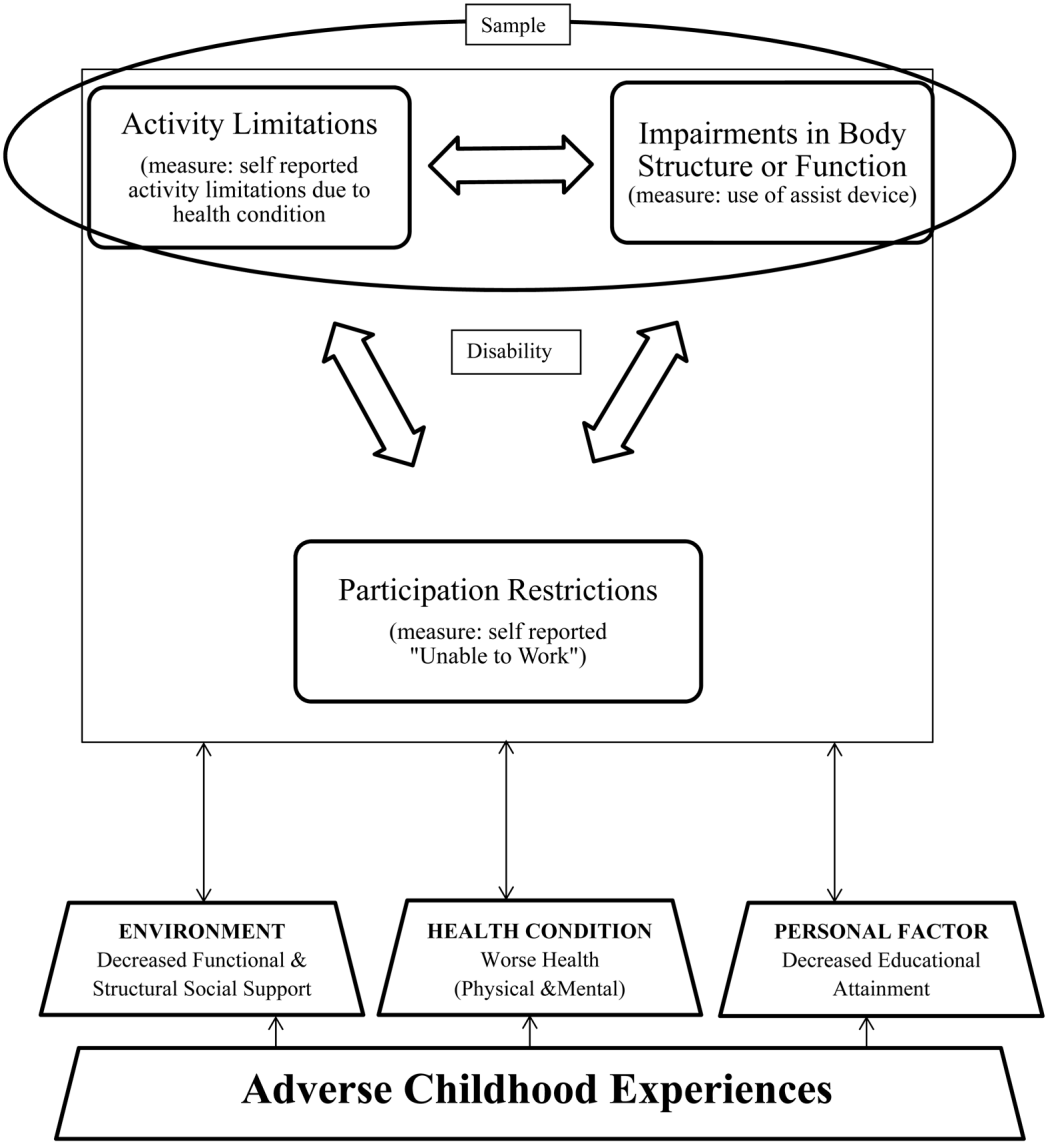
Conceptual Model.

To evaluate how ACEs might influence self-perceived ability to work, we used concepts from the International Classification of Functioning, Disability and Health (ICF). The ICF framework conceptualizes disability as comprised of one or more of the following components: impairments of body structures and functions, activity limitations, and participation restrictions. Contextual factors (personal and environmental factors) also influence disability in the ICF conceptual model [13, 14]. Not all those with impairments and/or activity limitations will have participation restrictions (such as work inability). For those with activity limitations, ability to work (participation) could be influenced by contextual factors and health condition-related impairments (Fig 1). Despite evidence that ACEs affect adult mental and physical health and personal factors such as health risk behaviors, less attention has been paid to how ACEs affect environmental factors such as support and to what extent support is a protective factor for work participation by people with health-condition related activity limitations. Receipt of adequate support can help to buffer the effects of life adversity [15]. Deficits in support have been associated with poorer health outcomes [16, 17] and decline in functioning [18].

Model of how ACEs may affect inability to work based on the International Classification of Functioning, Disability and Health constructs [14]. ACEs may lead to decreased functional and structural support (an environmental factor), worse adult health and decreased educational attainment (personal factors). All of these may impact participation restrictions (which include inability to work—our primary outcome).

Our first aim was to examine adverse childhood experiences (including category, type and number of categories) as a risk factor for work inability among participants with disability in a population-based survey. We hypothesized that as the number of categories of self-reported ACEs increased, the prevalence of self-reported work inability would increase.

Our second aim was to examine the relationship between ACEs and support which was conceptualized as having two components (structural and functional). We hypothesized that high ACEs exposure is a risk factor for poor support in adulthood. Specifically we hypothesized that as the number of ACE categories experienced increased the percentage with worse functional and structural support would also increase. We also hypothesized that better functional and structural support in adulthood would be protective against work inability in adulthood and that ACEs would have an effect of work inability that is independent of their effect on support.

For our third aim we wished to understand the effects of ACEs on work inability while accounting for current health status and educational attainment. ACEs are associated with lower educational attainment [9, 19] and worse physical and mental health in adulthood [5, 20–22]. In addition, both lower education and poor health are associated with work inability [23–25]. Thus we wished to evaluate whether ACEs have an effect on work inability independent of their effect on health status and education. We hypothesized that ACEs would have an independent effect.

For our fourth aim, we evaluated the effect of support on work inability while controlling for ACEs, education and health. We hypothesized that support would have an independent effect on work inability. Furthermore we hypothesized that good support may buffer the effects of poor health and be protective against work inability.

## Materials & Methods

We had previously confirmed with the University of Pennsylvania Institutional Review Board that research with the Behavioral Risk Factor Surveillance System’s publicly available de-identified data was not considered human subjects research (per the US federal definition).

### Data Source

We used data from the 2009 and 2010 Behavioral Risk Factor Surveillance System (BRFSS). The BRFSS is a joint project between the Centers for Disease Control and Prevention (CDC) and state health departments which surveys adults (age 18 and older) about their health risk factors and health [26]. In 2009 and 2010, the BRFSS used a disproportionate stratified sampling strategy to identify random households with landline telephones. Post-stratification adjustment of survey weights was performed to ensure a state’s sample demographics reflected its demographic distributions [27, 28]. The survey contained core component questions (demographics, employment, days of poor health, disability status, perceived emotional/social support) used by all U.S. states and territories. It also used optional modules which were incorporated into the survey at the discretion of each state/territory. The ACE module was an optional module used by fourteen states and the District of Columbia in 2009 and/or 2010 [26]. The ACE Module questions (Table 1) were adapted from a prior study on the effects of ACEs [29].

**Table 1:** Adverse Childhood Experience (ACE)^1^ Prevalence Estimates by Category, Type and ACE Score (Sample n = 13,009).

Category	Raw n	Weighted % (95% CI^2^)
**Abuse Categories**		
**1. Sexual Abuse**	3130	22.5 (20.9–24.1)
“How often did anyone at least 5 years older than you or an adult:—ever touch you sexually, -try to make you touch them sexually, -force you to have sex? (asked as three separate questions—an answer of once or more than once to any of the three questions was coded as yes).		
**2. Physical Abuse**	3624	28.1 (26.4–29.8)
“Before age 18, how often did a parent or adult in your home ever hit, beat, kick, or physically hurt you in any way? Do not include spanking. Would you say—never, once, more than once?" (once or more than once coded as yes)		
**3. Emotional Abuse**	5666	42.8 (40.9–44.6)
"How often did a parent or adult in your home ever swear at you, insult you or put you down?" Never, Once, More than once. (More than once was coded as yes)		
**Family Dysfunction Categories**		
**4**. **Domestic violence**	3202	25.2 (23.5–26.8)
"How often did your parents or adults in your home ever slap, hit kick punch or beat each other up?" (answers of once or more than once were coded as yes)		
**5. Mental Illness**	3831	28.7 (26.9–30.4)
"Did you live with anyone who was depressed, mentally ill or suicidal?"		
**6. Substance abuse**	5096	38.5 (36.7–40.4)
“Did you live with anyone who was a problem drinker or alcoholic?” and “Did you live with anyone who used illegal street drugs or who abuse prescription medication?” (an affirmative answer to any one of these two questions was counted as yes)		
**7. Criminal behavior**	1023	11.1 (9.7–12.5)
“Did you live with anyone who served time or was sentenced to serve time in a prison, jail or other correctional facility?”		
**8. Divorce or separation**	3662	31.1 (29.3–33.0)
Were your parents separated or divorced?		
**ACE Type**		
Abuse & Family Dysfunction	5599	41.7 (39.8–43.5)
Abuse Only	1498	11.3 (10.0–12.5)
Family Dysfunction Only	2477	19.5 (18.0–21.0)
**ACE Score (Number of ACE Categories Reported)**		
0	3435	27.6 (25.9–29.3)
1	2554	18.6 (17.2–20.0)
2	1988	14.6 (13.3–15.9)
3	1553	11.3 (10.1–12.6)
4 or more	3479	27.9 (26.1–29.6)

^1^The ACE module was administered in 5 states in 2009 (Arkansas, Louisiana, New Mexico, Tennessee, and Washington) and 10 states (Hawaii, Maine, Nebraska, Nevada, Ohio, Pennsylvania, Utah, Vermont, Washington and Wisconsin) and the District of Columbia in 2010.

^2^CI: Confidence Interval

### Sample

The state survey cooperation rates (percentage of those contacted who participated) ranged from 68.9 to 82.4. In addition, the Council of American Survey Research Organizations (CASRO) response rates (percentage of the estimated eligible who participated) ranged from 47.0 to 68.7 [27, 30]. Of the 89,810 survey respondents, we limited our sample to respondents younger than age 65 who affirmed one or both of the BRFSS disability questions (*n* = 14,983): “Are you limited in any way in any activities because of physical, mental or emotional health problems?” and “Do you now have any health problem which requires you to use special equipment, such as a cane, a wheelchair, a special bed, or a special telephone?” [31]. Of these, 652 were missing all ACE questions (97% of these had only partially completed the BRFSS survey). Of the remaining 14,331, an additional 1,322 cases were removed due to missing data (ACE: 536 cases; functional support: 124 cases; employment: 42 cases; physical and/or mental health: 458 cases; race: 139 cases; education: 10 cases; household member: 13 cases). Our final sample size was 13,009.

### Study Variables

The presence of ACEs was measured by questions regarding adverse childhood experiences occurring prior to age 18. The module included questions about sexual, physical and emotional abuse. It also contained questions about family dysfunction which included domestic violence, substance abuse, mental illness, and family member incarceration (Table 1). A total ACE score was created by summing the number of endorsed ACE categories (range 0–8). ACEs were also categorized by type: family dysfunction only, abuse only, both abuse and family dysfunction and none. Participants who chose, “Unable to work” as an answer to the question on employment status [31] were considered to have work inability.

Functional support was determined by the question, “How often do you get the emotional or social support that you need?” The variable was dichotomized into good (“Always,” “Usually”) versus poor (“Sometimes,” “Rarely,” or “Never”) functional support [31]. Good structural support was defined as living with another adult in the household. Educational attainment was divided into 4 categories (less than high school, high school graduate, some college/technical school, college/technical school graduate). Poor physical and mental health were measured by the self-reported number of days of poor physical and mental health in the past thirty days (range 0–30) respectively [31].

### Analysis

Descriptive and logistic regression analyses were conducted with SAS^®^ 9.3 (SAS Institute, Inc., Cary, NC, 2011). The complex survey design specifications and survey weights were used in all analyses.

We first performed the following descriptive analyses: 1. Prevalence of ACEs. 2. Demographics characteristics and prevalence of ACEs, and work inability in relation to these characteristics. 3. Inability to work, functional and structural support by ACE category, type and score. (We added logistic regression to this analysis to evaluate the relationship while controlling for demographic variables). 4. The prevalence of four or more ACEs and work inability by support, educational attainment and past month physical and mental health.

Demographic characteristics included age, sex and race. These variables were considered potential confounders. Age, in particular, was known to be a negative confounder from prior work [12]. Based on our conceptual model (Fig 1), education and health were considered potential intermediate variables on the causal pathway between ACEs and inability to work. They were treated as other important predictors. Although support was considered to be influenced by ACEs, we also evaluated whether the effect of support varied according to health status. All variables were chosen a priori based on our conceptual model. No additional variables were evaluated.

We then evaluated whether ACEs had an independent effect on work inability after adjusting for contextual factors and health individually. This involved 5 separate logistic regression models: ACEs and functional support, ACEs and structural support, ACEs and education, ACEs and physical health, ACEs and mental health with all models also including the demographic variables (age, sex, and race).

After confirming ACEs had an independent effect in these bivariate models, we then performed a series of logistic regression analysis starting with ACEs only (1) then adding the support variables (2), followed by education (3), and then health (4). Age, gender, and race/ethnicity were included as covariates in all of the multivariate analyses. An odds ratio (OR) whose 95% confidence interval (CI) did not cross one was considered statistically significant (α = 0.05). Although, in descriptive analyses, age was reported categorically, it was entered as a continuous predictor in our logistic regression analyses.

We initially evaluated the ACE Score as a categorical variable, since model fit seemed better using ACE as a categorical variable compared to a continuous variable. However after finding that there was not a significant difference between ACEs 0–3 and that there were wide and overlapping confidence intervals (due to smaller numbers) at higher levels of ACEs, we dichotomized ACE Score into 4 or more/less than 4 categories for our multivariate analyses examining the effect of health and contextual factors.

Days of poor mental and physical health were initially modeled continuously. However, these variables had a non-uniform distribution with peaks at 30 days and 0 days with sparse data in between. There was violation of the logistic regression assumption of a linear relationship between a continuous predictor and the log odds. Thus, we also modeled these variables as categorical variables, using three categories: good (3 days or fewer), intermediate (4–15 days) and poor (16 or greater days of poor health). We also evaluated potential nonlinear transformations of the health variables using spline functions. The choice of how to model days of poor health did not affect the odds ratios (ORs) of ACEs, support or education. Therefore, we chose to present the categorical results, as these are more interpretable. In particular, the health categories were particularly important for the analysis of whether the effect support on work inability varied by level of health. The choice of categories was made prior to analysis. Although days of poor health are dichotomized in other studies [25], having at least three categories made sense in terms of the outcome measure of work inability. We included up to 3 days of poor health in the good health category with the rationale that even those in good health may have a few days poor health due to a minor illness and that these may not interfere with employment. Having poor health for over half the month was considered likely to have a strong effect on ability to work.

We evaluated possible differential effects of support on inability to work by health status by stratifying by physical and mental health categories separately. We also tested whether there were statistically significant differences between groups by adding an interaction term to our final model. Where there appeared to be a substantial variation of effect by strata, we also modeled the joint effect against a common reference [32]. We assessed multiplicative interaction, which was considered most appropriate for our data [33, 34].

## Results

ACEs were common, with 72.4% of participants having experienced at least one ACE category and 27.9% having experienced four or more categories. The most commonly reported ACEs were emotional abuse (42.8%) and living with a substance abusing parent/caregiver (38.5%). The least common ACE was having an incarcerated caregiver (11.1%) Over forty percent of participants had experienced both abuse and family dysfunction categories. (Table 1).

Table 2 shows the demographic distribution of the sample along with the prevalence of ACEs and work inability in each group. Of the 13,009 respondents, 51.9% were female, and the majority of respondents (81.9%) identified as non-Hispanic white (Table 2). Over forty percent of the youngest age group (ages 18–29) reported experiencing at least four ACE categories which was over twice the rate of the oldest group (ages 55–64). The oldest two groups (45–64) had almost twice the rate of being unable to work than the youngest group. (Table 2) Although in the overall prevalence of work inability was 15.8% in youngest age group; in young adults reporting a high ACE burden, the prevalence was 27.4% (95% CI 15.7–39.1) versus 6.7% (3.2–10.2) for those with lower ACE exposure.

**Table 2:** Sample Demographics: Relationship to ACEs^1^ and Perceived Work Inability.

Characteristic	Raw n	Weighted (wt.) % of sample (n = 13,009)	ACE ≥ 4 (wt. %)	Unable to Work (wt. %)
**Age**			p<0.0001	p<0.0001
18–29	609	11.3	43.6 (35.9–51.3)	15.8 (9.9–21.6)
30–44	2248	31.3	31.5 (27.9–35.1)	20.5 (17.4–23.6)
45–54	4017	28.6	27.2 (24.5–29.9)	30.6 (27.8–33.4)
55–64	6135	28.8	18.3 (16.5–20.2)	28.1 (25.8–30.3)
**Sex**			p<0.0001	p = 0.10
Male	4897	48.1	22.6 (19.8–25.3)	23.7 (21.1–26.2)
Female	8112	51.9	32.8 (30.5–35.0)	26.3 (24.4–28.3)
**Race**			p<0.0001	p<0.0001
Non-Hispanic White	10242	81.9	26.8 (24.8–28.8)	23.7 (21.9–25.4)
Non-Hispanic Black	1017	8.5	28.6 (23.1–34.0)	34.2 (28.9–39.5)
Hispanic	647	3.8	31.2 (24.7–37.7)	25.0 (19.3–30.8)
Asian/Nat Haw/PI	272	1.3	10.7 (4.4–17.0)	10.2 (5.4–15.0)
Other^2^	831	4.6	47.2 (39.4–55.0)	36.9 (28.6–45.1)

^1^Adverse Childhood Experiences

^2^Other includes Native American, Multiracial, and Other

Table 3 explores the relationship between ACEs and work inability and support. For all ACE categories, the prevalence of work inability was higher for those having experienced the category than for those who had not. The prevalence of work inability for those who experienced a particular ACE category was close to 30% for most categories. The prevalence of work inability was over 10% higher in those experiencing the family dysfunction ACE categories (over 27%) compared to those experiencing abuse only ACE categories (16.6%) Participants experiencing 4 or more ACE categories had a work inability prevalence of 31.4% which was over 10% higher than the prevalence of those who had not experienced any ACEs. Compared to those reporting no ACEs, the adjusted odds (95% confidence interval) of work inability of those with an ACE score ≥ 4 was [OR 1.91 (1.55–2.44), p <0.0001] (Table 3).

**Table 3:** Relationship between Adverse Childhood Experience (ACE) Categories, Type and Score with Perceived Inability to Work and Support.

	Unable to Work		Structural Support: Only Adult in Household		Functional Support: Sometimes, Rarely, Never	
	Weighted (wt.) row %	Adjusted^1^ OR (95% CI^2^)	wt. row %	Adjusted^1^ OR (95% CI)	wt. row %	Adjusted^1^ OR (95% CI)
**ACE Categories**^3^						
Sexual Abuse						
No	23.1	reference	17.7	reference	27.2	reference
Yes	31.6	1.6 (1.3–1.9)	21.4	1.3 (1.1–1.5)	42.9	2.0 (1.7–2.5)
Physical Abuse						
No	23.8	reference	17.3	reference	25.2	reference
Yes	28.2	1.3 (1.1–1.6)	21.7	1.4 (1.2–1.7)	44.9	2.4 (2.0–2.9)
Emotional Abuse						
No	23.9	reference	17.1	reference	23.3	reference
Yes	26.6	1.2 (1.0–1.5)	20.5	1.4 (1.2–1.6)	40.7	2.3 (1.9–2.7)
Domestic Violence						
No	22.9	reference	17.6	reference	27.5	reference
Yes	31.3	1.5 (1.2–1.8)	21.4	1.3 (1.1–1.5)	40.5	1.7 (1.4–2.1)
Mental Illness						
No	23.3	reference	17.7	reference	27.0	reference
Yes	29.3	1.5 (1.3–1.9)	20.7	1.4 (1.2–1.6)	40.2	1.8 (1.5–2.2)
Substance Abuse						
No	22.4	reference	17.8	reference	25.3	reference
Yes	29.2	1.5 (1.3–1.8)	19.8	1.2 (1.0–1.4)	39.5	1.9 (1.6–2.2)
Criminal Behavior						
No	24.2	reference	18.7	reference	29.2	reference
Yes	31.4	1.7 (1.2–2.3)	17.6	1.0 (0.8–1.4)	43.4	1.7 (1.2–2.2)
Divorce/Separation						
No	23.9	reference	19.1	reference	27.6	reference
Yes	27.6	1.3 (1.1–1.6)	18.3	1.1 (1.0–1.3)	37.7	1.5 (1.2–1.8)
**ACE Type**^4^						
Abuse & Family Dysfunction	28.8	1.6 (1.3–2.0)	21.0	1.6 (1.3–1.9)	40.8	3.1 (2.5–3.9)
Abuse Only	16.6	0.8 (0.6–1.1)	16.6	1.1 (0.9–1.5)	29.4	2.0 (1.4–2.7)
Family Dysfunction Only	27.4	1.4 (1.1–1.8)	18.5	1.2 (1.0–1.6)	28.5	1.8 (1.3–2.3)
**ACE Score**						
0	21.0	reference	15.7	reference	17.7	reference
1	22.2	1.1 (0.8–1.4)	17.4	1.2 (0.9–1.4)	25.9	1.6 (1.2–2.1)
2	25.4	1.3 (1.0–1.7)	19.6	1.3 (1.1–1.7)	30.7	2.0 (1.5–2.7)
3	23.5	1.2 (0.9–1.6)	18.8	1.3 (1.0–1.7)	36.2	2.6 (1.9–3.5)
4 or more	31.4	1.9 (1.5–2.4)	21.6	1.7 (1.4–2.1)	44.7	3.7 (2.9–4.7)

^1^All models adjusted for age, sex and race

^2^CI: Confidence Interval

^3^Logistic regression reference for ACE categories is no experience in that category. Each category modeled separately

^4^Reference for ACE type is no ACE (Single model).

The association between ACEs and functional support appeared stronger than between ACEs and structural support (Table 3). For each ACE category, around 40% of those who had experienced the category reported poor functional support (range 38–45%). This percentage was universally higher than the percentage of those who had not experienced the category. The percentage of those reporting poor functional support increased from 17.7% (0 ACEs) to 44.7% (4 or more ACE categories). In contrast, there was a smaller increase in percentage reporting poor structural support as ACEs increased (from 15.7% (0 ACEs) to 21.6% (4 or more ACE categories) (Table 3).

Overall reports of poor functional support (30.8%) were more common than reports of poor structural support (18.0%) (Table 4). Both poor structural support and poor functional support were associated with work inability. Almost 40% of those with poor functional support and 34.5% of those with poor structural support reported work inability compared to 21.7% and 20.8% of those reporting good structural and functional support respectively (Table 4).

**Table 4:** Support, Recent Health and Education and Relationship to ACEs and Work Inability.

	Raw n	Weighted (wt.) Column %	4 or More ACEs wt. Row % (95% CI^1^)	Unable Work wt. Row % (95% (CI))
**Structural Support**				
Poor	4605	18.6	32.4 (29.6–35.1)	39.6 (36.7–42.5)
Good	8404	81.4	26.8 (24.8–28.9)	21.7 (19.9–23.5)
**Functional Support**				
Poor	3891	30.8	40.5 (36.9–44.1)	34.5 (31.2–37.8)
Good	9118	69.2	22.2 (20.3–24.1)	20.8 (19.1–22.5)
**Education**				
< High School	1049	9.8	40.7 (33.8–47.5)	47.4 (40.7–54.1)
High School Graduate	3618	31.5	30.2 (26.8–33.6)	30.8 (27.7–33.8)
Some College	4281	32.1	29.0 (26.0–32.0)	24.9 (22.0–27.8)
College graduate	4061	26.7	19.0 (16.4–21.5)	10.2 (8.4–12.0)
**Physical Health**				
Poor (16–30 days)	3712	27.2	33.4 (30.0–36.8)	48.5 (45.0–52.0)
Intermediate (4–15 days)	3057	23.8	30.6 (27.0–34.2)	25.5 (22.3–28.7)
Good (3 or fewer days)	6240	49.0	23.5 (21.0–25.9)	11.8 (10.1–13.5)
**Mental Health**				
Poor (16–30 days)	2679	22.2	44.2 (40.0–48.3)	43.1 (39.1–47.2)
Intermediate (4–15 days)	2839	22.4	32.4 (28.7–36.0)	26.2 (23.0–29.4)
Good (3 or fewer days)	7491	55.4	19.5 (17.4–21.6)	17.3 (15.6–19.1)

^1^Confidence Interval

Education and health were strongly associated with ACEs and work inability. The prevalence of ACEs decreased with increasing educational attainment going from 40% in those with less than a high school education to 19.7% of college graduates. The decline in work inability prevalence was even greater. Almost half of those with less than a high school education reported work inability compared to only 10% of college graduates (Table 4).

Almost half the sample reported 3 or fewer days of poor physical health and over half reported 3 or fewer days of poor mental health. The prevalence of work inability was lower in those reporting good physical health (11%) than those reporting good mental health (17%). The percentage reporting work inability was substantially higher in those reporting poor health over half the month. It was close to 50% for those with poor physical health and 44% of those reporting poor mental health (Table 4).

### Logistic Regression Analyses

Logistic regression results indicated that those with an ACE score ≥ 4 had a higher adjusted odds ratio (95% confidence interval) of work inability [OR 1.74 (1.43–2.10), p <0.0001] compared to those with ACE score less than four, adjusting for age, sex and race. ACE burden was an independent predictor of work inability in the following regression models (all adjusted for age, sex and race): ACE and functional support, ACE and structural support, ACE and education, ACE and physical health and ACE and mental health.

The results of the sequential logistic regression analyses are shown in Table 5. ACEs appeared to have an independent effect on inability to work after adjusting for support (Model 2) and support and education (Model 3). After health was added (Model 4), the OR of four or more ACEs (compared to fewer than four) was closer to one and no longer statistically significant. A similar result was seen with functional support. Structural support, however, still had a substantial protective association with work inability (OR 0.50 (0.41–0.60)). Higher education was highly protective against work inability and the odds of work inability decreased as educational attainment increased. Physical and mental health were independent predictors of work inability (Table 5).

**Table 5:** Multivariate Logistic Regression^1^ Analyses of Odds of Inability to Work.

	Model 1: ACE^2^	Model 2: ACE, and Support	Model 3: ACE, Support, Education	Model 4: Model 3 Plus Health
Variable	aOR^3^ (95% CI^4^)	aOR (95% CI)	aOR (95% CI)	aOR (95% CI)
**ACE ≥ 4** (reference: ACE < 4)	1.74 (1.43–2.10)*	1.51 (1.24–1.85)*	1.40 (1.13–1.72)**	1.18 (0.95–1.47, p = 0.13)
**Good functional support** (reference: poor)	***	0.55 (0.46–0.67)*	0.66 (0.54–0.81)*	0.88 (0.71–1.08, p = 0.22)
**Good structural support** (reference: poor)	***	0.51 (0.43–0.61)*	0.49 (0.41–0.58)*	0.50 (0.41–0.60)*
**Education**				
Less than High school	***	***	reference*	reference*
High School Graduate	***	***	0.52 (0.38–0.71)	0.56 (0.41–0.78)
Some College	***	***	0.38 (0.28–0.52)	0.41 (0.30–0.57)
College Graduate	***	***	0.14 (0.10–0.19)	0.18 (0.13–0.25)
**Physical Health**^5^				
Good	***	***	***	reference*
Intermediate	***	***	***	2.11 (1.64–2.72)
Poor	***	***	***	4.58 (3.61–5.80)
**Mental Health**^5^				
Good	***	***	***	reference*
Intermediate	***	***	***	1.31 (1.04–1.66)
Poor	***	***	***	1.97 (1.54–2.53)

^1^All Models controlled for age, sex and race

^2^ACE = Adverse Childhood Experiences

^3^aOR = adjusted odds ratio

^4^CI = confidence interval

^5^Days of poor health: Good: 3 or fewer; Intermediate: 4–15; Poor: 16–30.

*p<0.0001

**p < 0.001

***Not in Model

In the stratified logistic regression analysis by physical health status (adjusting for ACEs, demographics, education and mental health), functional support was protective in those with good physical health (OR 0.66 (0.46–0.95)), but not for those with intermediate (OR 1.15 (0.76–1.75)) or poor physical health (OR 0.92 (0.67–1.26)). Since there was no evidence of any protective effect at intermediate and poor physical health, we combined these levels for the logistic regression analysis using a common reference and the separate analysis evaluating an interaction term (Table 6). These analyses also supported that functional support was only protective for those with good physical health. In contrast, when performing logistic regression stratified by mental health, functional support was not protective at any level of mental health.

**Table 6:** Evaluation of the Differential Effect of Support at Different Levels of Health^1^.

	aOR^2^ (95% CI^3^)	aOR (95% CI); p-value	Within Strata aOR (95% CI)
	**Poor Functional Support**	**Good Functional Support**	**Good Compared to Poor Functional Support by Health Strata**
**Good Physical**^4^ **Health**			
** No**	reference	1.02 (0.80–1.31)	0.97 (0.76–1.25)
** Yes**	0.30 (0.20–0.45)	0.19 (0.14–0.25)	0.66 (0.46–0.95)
Interaction Term			*Ratio of aORs*: *0*.*62 (0*.*40–0*.*97); p = 0*.*038*
**Good Mental**^5^ **Health**			
** No**	reference	0.86 (0.66–1.13)	0.89 (0.61–1.28)
** Yes**	0.64 (0.44–0.93)	0.53 (0.40–0.70)	0.85 (0.65–1.10)
Interaction Term			*Ratio of aORs*: *0*.*96 (0*.*63–1*.*47); p = 0*.*85*
	**Poor Structural Support**	**Good Structural Support**	**Good Compared to Poor Structural Support by Health Strata**
**Good Physical**^4^ **Health**			
** No**	reference	0.54 (0.43–0.68)	0.53 (0.42–0.66)
** Yes**	0.26 (0.19–0.36)	0.11 (0.08–0.15)	0.41 (0.30–0.57)
Interaction Term			*Ratio of aORs*: *0*.*80 (0*.*55–1*.*18); p = 0*.*17*
**Good Mental**^5^ **Health**			
** No**	reference	0.61 (0.47–0.78)	0.59 (0.46–0.75)
** Yes**	0.71 (0.51–0.98)	0.28 (0.20–0.37)	0.39 (0.30–0.52)
Interaction Term			*Ratio of aORs*: *0*.*66 (0*.*46–0*.*95); p = 0*.*026*

^1^ACEs, demographics, education and support were in all models. Only one interaction term was tested at a time

^2^aOR: adjusted OR

^3^CI: Confidence Interval

^4^Physical health (3 category) was in all models stratified by mental health or containing a mental health (binary)/support interaction term

^5^Mental health (3 category) was in all models stratified by physical health or containing a physical health (binary)/support interaction term

In contrast, while structural support was protective at all three levels of physical health, the odds ratios did not differ significantly between levels of physical health. The protective effects of structural support, however, were stronger for those with good mental health: OR (95% CI) 0.39 (0.30–0.51) for good; OR 0.58 (0.41–0.83) for intermediate and OR 0.63 (0.44–0.89) for poor mental health. This was confirmed again through the evaluation of an interaction term and logistic regression using a common reference (Table 6).

## Discussion

The prevalence of ACEs was strikingly high in our sample and strongly associated with decreased functional support, lower educational attainment and worse physical and mental health. As hypothesized, participants experiencing high levels of childhood adversity had a higher odds of work inability compared to those that had not. The effect, however, appeared to be more of a threshold effect as opposed to a graded effect and was strongest in the youngest age group. Functional and structural support were protective against work inability in adults with self-reported disability. After adding education and recent health to the model, there no longer was an independent effect of ACEs or functional support on work inability. Structural support, however, remained strongly protective. ACEs did appear to have an independent effect on work inability in the youngest age group. Functional support appeared to be protective in those with good physical health.

The finding of increased rates of work inability in those reporting ACEs is in line with previous research that has connected ACEs with worse worker performance [7], functional impairment in role performance (including work, social life, intimate relationships and household) [6, 35], lower levels of employment [9, 36, 37], and increased rates of employment disability [38]. Childhood abuse has been associated with lower self-efficacy [39], an important predictor of returning to work after a long-term absence due to illness [40]. It also has been associated with decreased executive functioning in middle adulthood [41], which is important for obtaining and maintaining employment. Furthermore, ACEs are associated with increased risk of experiencing physical or sexual violence in adulthood [42]. Prior research about women with disabilities has found that women who have experienced prior physical or sexual abuse or intimate partner violence have higher unemployment rates [43, 44]. Finally, childhood adversity is strongly associated with increased engagement in health risk behaviors such as drug abuse [11, 20, 45, 46], which clearly can interfere with ability to work.

Our finding that the percentage of adults reporting good functional support decreased steadily as the number of self-reported ACE categories increased is also consistent with the literature. Previous research has suggested that childhood maltreatment, such as physical/sexual abuse and neglect, may negatively impact an adult’s ability to create and maintain healthy relationships [47]. Further, childhood adversity can have negative effects on intimate relationships [47] and social network size [48].

Our hypotheses regarding the independent effect of ACEs and support on work inability, after inclusion of other important predictors, were only partially supported. This finding was not unexpected given the strong associations between childhood adversity and worse physical and mental health in adulthood [5, 10, 11, 20, 22, 49]. Childhood adversity is also associated with increased disorder-specific functional impairment [35] and disability [12]. The effects of poor physical and mental health are intertwined and comorbid mental health conditions are an important contributor to role impairment in those with physical health conditions [45, 50, 51].

Our hypotheses regarding the relationship between support and inability to work were only partially supported. Only structural support had an independent effect after controlling for education and health. In contrast to our original hypothesis, functional support appeared to have a protective association with work inability only in those reporting good physical health but not in those reporting intermediate or poor physical health.

### Limitations

The survey relied on landlines only and primary cellphone users were not included in the sample, which may affect generalizability.

All measures were obtained at a single time-point and thus our findings represent associations. The cross-sectional nature also precluded us from testing formal theories of mediation (such as evaluating health as a mediator and looking at the direct and indirect effects of ACEs on work inability). Although evaluating for the presence of alternative effect pathways by controlling for an intermediate variable is a common epidemiological method [52], because of the correlations between of our major predictors, it is possible that the change in coefficient and loss of significance in Model 4 is due to multicollinearity as opposed to evidence of lack of an independent effect of ACEs (other than their effect on education and health). Also while structural support appeared to have a strong independent effect, it is possible this is due to residual uncontrolled confounding. While we treated support as a predictor of inability to work, is also possible that inability to work affects support. For our underlying predictor, ACEs, however, the possibility of reverse causation is less of an issue, since present day work inability cannot cause ACEs.

The study relied on retrospective self-reports of ACEs which have reasonable validity and tend to be relatively stable over time, but are prone to underreporting bias [47, 48, 53–57]. The number of questions used for each measure was limited, precluding a more in depth understanding of the answers. To address the possibility that those reporting poor functional support may not have sought or required support, as opposed to not having the support available, we included a measure of structural support [58].

The BRFSS definition of disability is broad [59] and information about the details and severity of the disability is not available. A more detailed measure would allow for more in-depth exploration of the association between specific ACEs and specific components of disability.

We do not know the reasons why people felt they were “unable to work.” However, the self-reported prevalence of work inability among respondents under age 65 without self-reported disability was 1% in the full BRFSS sample versus 25% in our disability sub-sample. Hence, it is likely that disability plays a large role in ability to work. Similarly, we may have underestimated the percentage of people whose disability prevents them from working by focusing solely on those who responded they are “unable to work.” Previous research has demonstrated that ACEs are associated with early disability-related retirement [60], but we could not assess for this possibility with these data.

## Conclusions

This study helps expand the literature of risk and protective factors that may affect work inability in adults with disability. The impact of ACEs on multiple adverse adult outcomes including health risk behaviors, health, education, disability and work inability highlights the importance of interventions to prevent and/or mitigate the effects of ACEs as early as possible [4, 5, 9, 61]. Mitigation efforts should include research on ways to improve educational attainment in children, adolescents and young adults with known ACE exposures.

However, given high disability rates in the United States, an understanding of the factors that affect the ability to work of adults with disability is critical to designing effective interventions. Enabling greater numbers of adults with disability to work not only has clear societal economic advantages, but also provides individual level benefits such as increased self-esteem, greater independence and social inclusion.

Our study has implications for practice and future research. It is important to realize that a greater proportion of adults with a disability experienced ACEs and over twice as many have a high ACE burden (four or more ACEs) compared to adults without a disability [4]. In our study, ACEs appeared to be more strongly associated with work inability in the youngest age group which also had the highest prevalence of ACEs. Effectively addressing the effects of ACEs on work ability in this group may be particularly beneficial given that this age group could have as many as 30–40 productive work years ahead of them.

Even if the effects of ACEs on work inability is more indirect such as through the effect of ACEs on health, it may still be important to address the impact of ACEs in these individuals in order to improve health. There is evidence that one needs to take ACE exposure into account to effectively address health risk behaviors and certain health problems in adults who have experienced ACEs and that ACE status may have implications for choice of treatment [62–65].

Assessing for ACEs and their effect on current health and support can guide avenues for treatment (e.g. trauma therapy, social skill development). It is reasonable to suggest that a clinician should be aware of these factors and work towards preventative measures to decrease future risk of work inability. Preventive measures can include exploring a patient’s perception of available social support, assistance with identifying potential sources of support (e.g. family, friends, available support groups), and help for the patient to increase his or her social ability and currency. The strong protective effect of education suggests that policies supporting vocational rehabilitation, and research on the most effective educational interventions for those with disability are vital [66].

Continued research that refines our understanding of risk and protective factors associated with the ability of adults with a disability to work is needed [67]. Longitudinal studies in adults with disability that evaluate the effect of ACEs on outcomes such as participation are particularly important. As the field advances, specific knowledge related to the relationship between ACEs, mental and physical health, external factors, and genetic predispositions will facilitate the development of targeted treatments to enable all adults with disability reach their full employment and participation potential.
